# Pelvic obliquity and measurement of hip displacement in children with cerebral palsy

**DOI:** 10.1080/17453674.2018.1519104

**Published:** 2018-10-17

**Authors:** Gunnar Hägglund, Mikael Goldring, Maria Hermanson, Elisabet Rodby-Bousquet

**Affiliations:** 1 Department of Clinical Sciences, Lund University, Lund;; 2 Department of Orthopaedics, Skane University Hospital, Lund;; 3 Department of Surgery, Sahlgrenska University Hospital/Östra, Göteborg;; 4 Centre for Clinical Research, Uppsala University, Region Västmanland, Västerås, Sweden

## Abstract

Background and purpose — Pelvic obliquity, common in individuals with cerebral palsy (CP), changes the muscle force vector on the hip joint and probably affects the risk of hip dislocation. We evaluated a new method for measurement of hip displacement in CP that takes the pelvic obliquity into account: the pelvic adjusted migration percentage (PAMP).

Children and methods — From the Swedish surveillance program for cerebral palsy (CPUP), the first pelvic radiograph of 268 children <18 years in southern Sweden during a 3-year period were evaluated. Pelvic obliquity, PAMP, and the migration percentage (MP) were measured. 50 radiographs were randomly selected for analysis of interrater reliability by three raters using the intraclass correlation coefficient (ICC). The correlations between PAMP/MP and pelvic obliquity were analyzed with Pearson correlation coefficients.

Results — The interrater reliability for all 3 measurements was high (ICCs 0.88–0.97). The correlation between the high side of the pelvic obliquity and the difference between right and left hip displacement was higher for PAMP (r = 0.70) than for MP (r = 0.41).

Interpretation — The new PAMP measurement showed high interrater reliability and a higher correlation with pelvic obliquity than MP. We suggest the use of PAMP at least in hips with a pelvic obliquity exceeding 5°.

Hip displacement in cerebral palsy (CP) is caused by abnormal forces of the femoral head on the acetabulum (Kalen and Bleck 1985). Spasticity, muscle weakness, and/or poor positioning are the underlying causes of the altered magnitude and direction of the forces (Kalen and Bleck 1985, Miller et al. 1999).

In a computer modelling analysis, Miller et al. (1999) showed that in a normal hip the force of the hip muscles is directed to the superomedial part of the acetabulum. In a spastic hip the force is increased, and the imbalance causes an abnormal position of the hip in adduction and internal rotation. This ‘spastic position’ of the hip shifts the direction of forces to the posterosuperolateral aspect of the acetabulum. Because of the changed force direction, the femoral head is at risk for lateral displacement, usually measured as an increased migration percentage (MP) (Reimers 1980) (Figure 1a).

**Figure 1a.**
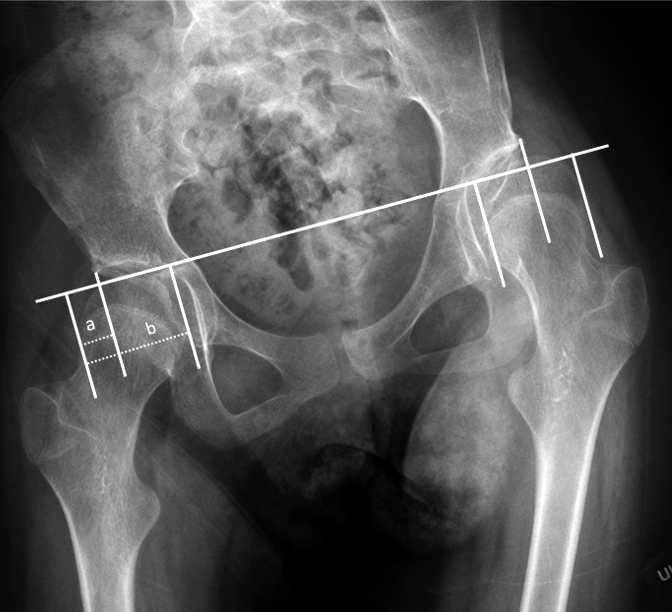
Pelvic obliquity in a 12-year-old girl with cerebral palsy in GMFCS level IV. Measurement of migration percentage (MP). MP = a/b × 100. MP =46% in the left hip and 32% in the right hip.

Pelvic obliquity (PO) is a common deformity in individuals with CP (Porter et al. 2007). PO can be measured as the angle between the horizontal plane and Hilgenreiner’s line, or a line between the acetabular teardrops, the lowest prominence of the ischial tuberosities, or the highest prominence of the iliac crest (Figure 1b). The hip on the high side of the PO is positioned in adduction with correspondingly more lateral direction of the muscle force vector. The acetabulum on the high side becomes more vertical, which uncovers the femoral head. The hip on the lower side is in abduction and the acetabulum is more horizontal with increased coverage of the femoral head.

**Figure 1b.**
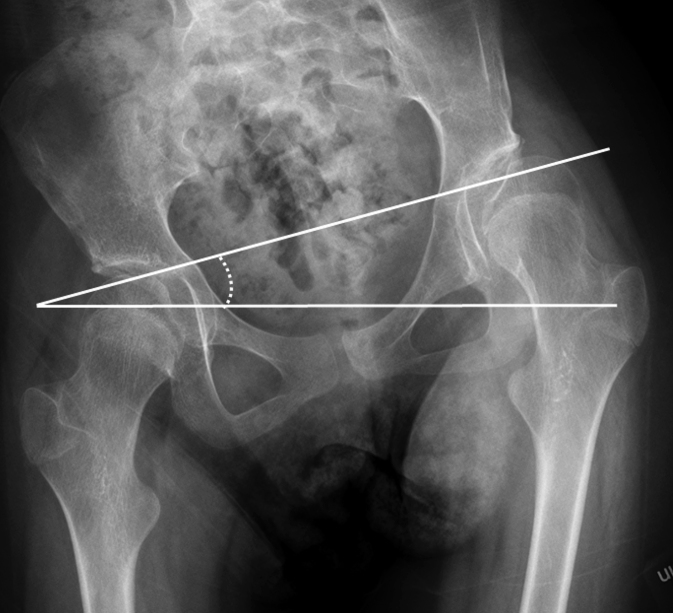
Measurement of pelvic obliquity (PO). PO =15°, left side elevated.

MP is the gold standard for measuring hip displacement and is used in most hip surveillance programs (Dobson et al. 2002, Hägglund et al. 2014). In MP, the lateral displacement is related to Hilgenreiner’s line. This means that the measurement of MP is not related to a PO.

Therefore, we evaluated a new measurement method of hip displacement that accounts for the PO, a pelvic adjusted migration percentage (PAMP), by relating the displacement to a horizontal line instead of Hilgenreiner’s line (Figure 1c).

**Figure 1c.**
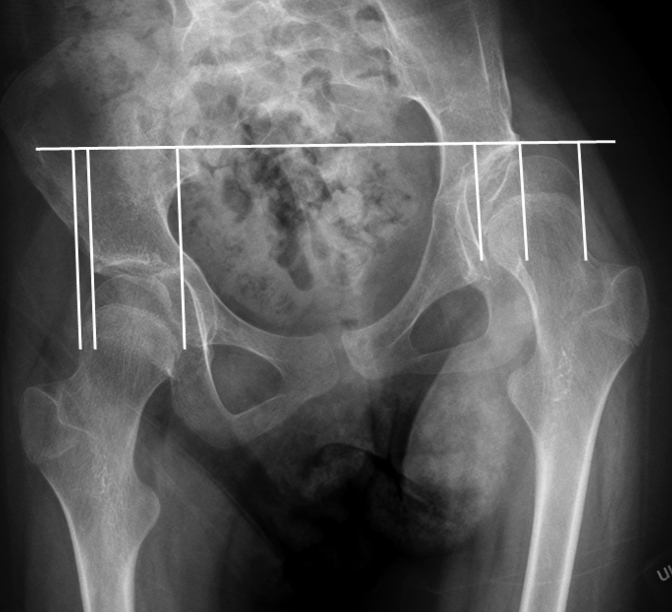
Measurement of pelvic adjusted migration percentage (PAMP). PAMP =59% in the left hip and 15% in the right hip.

## Children and methods

In CPUP—the Swedish surveillance program for CP—more than 95% of all children with CP are followed with standardized repeated examinations (Alriksson-Schmidt et al. 2017). The gross motor function is classified by the child’s physiotherapist according to the Gross Motor Function Classification System (GMFCS) (Palisano et al. 1997). GMFCS is an age-related, 5-level system in which children at level I are the least affected. CPUP also includes standardized repeated anteroposterior pelvic radiographic examinations (Hägglund et al. 2014). Children at GMFCS levels III–V are examined radiographically once a year up to 8 years of age and those at level II are examined at 2 and 6 years of age. Children at level I are not examined radiographically provided that the physiotherapist’s reports show a normal pain-free range of hip motion. After 8 years of age, the children are followed-up individually based on the previous radiographic and clinical findings.

In the present study, all radiographs of children <18 years in southern Sweden (Skåne and Blekinge with 1.4 million inhabitants) during the 3-year period from July 1, 2014 to June 31, 2017 were analyzed. In children with repeated radiographic examinations during the study period, the first radiograph was used. Children operated for scoliosis or with varus osteotomy of the proximal femur before the first examination were excluded.

On all radiographs, PO, MP, and PAMP were measured. The difference in hip displacement between the right and left hip, measured as both MP and PAMP, was analyzed in relation to the degree of PO.

50 radiographs were randomly selected for analysis of interrater reliability. 3 raters (GH, MG, MH) independently measured the PO, MP, and PAMP. All measurements were sent separately to a 4th blinded person (ERB) for statistical analyses.

## Statistics

Interrater reliability was evaluated using the intraclass correlation coefficient (ICC) and 95% confidence interval (CI) with a 2-way random model, and absolute agreement for single measures (McGraw and Wong 1996). Scatter plots with fitted values for the difference between right and left hip in hip displacement, measured as MP, PAMP, and PO, were prepared. Correlations between MP/PAMP and PO were investigated with Pearson correlation coefficients (r) and 95% bootstrap CIs were added based on 1,000 bootstrap samples. The result was interpreted according to Evans (1996); r = 0.00–0.19, negligible; 0.20–0.39, weak; 0.40–0.59, moderate; 0.60–0.79, strong; and 0.90–1.00, very strong correlation. The statistical analyses were performed using IBM SPSS Statistics version 24 (IBM Corp, Armonk, NY, USA).

## Ethics, funding, and potential conflicts of interest

The study was approved by the Medical Research Ethics Committee at Lund University (LU-443-99). The study was funded by Linnea and Josef Carlssons foundation and Stiftelsen för bistånd åt rörelsehindrade i Skåne. The authors declare no conflicts of interest.

## Results

During the 3-year period, 307 children (167 boys) were examined radiographically. Of these, 39 children were excluded: 32 children (17 girls) had been operated with varus osteotomy; 6 children (4 girls) had received surgery for scoliosis; and 1 girl was operated with both varus osteotomy and spine surgery before the first examination. The remaining 268 individuals were included in the analysis. The age at examination and GMFCS distribution are presented in Table 1.

**Table 1.** Distribution of age and Gross Motor Function Classification System (GMFCS) levels

Age (years)	GMFCS level				
	II	III	IV	V	Total
< 3	18	8	10	17	53
3–5	18	14	17	14	63
6–8	17	9	15	12	53
9–11	5	12	15	10	42
12–14	3	7	15	8	33
15–18	1	10	8	5	24
Total	62	60	80	66	268

There was high interrater reliability for all 3 measurements, with the lower levels of the 95% CI above 0.7 (Table 2). The highest ICC was seen for PO measurement (ICC 0.97). Measurement of PAMP showed slightly higher intraclass correlation coefficients for both the right and the left side (ICC 0.91; 0.93) than MP (ICC 0.88; 0.89).

**Table 2.** Interrater reliability estimated by intraclass correlation coefficient (ICC)

Factor	ICC	95% CI
Pelvic obliquity	0.97	0.96–0.98
MP right	0.88	0.78–0.94
MP left	0.89	0.78–0.94
PAMP right	0.91	0.83–0.95
PAMP left	0.93	0.86–0.96

MP: migration percentage;

PAMP: pelvic adjusted migration percentage;

p < 0.001 for all measurements.

**Figure 2.**
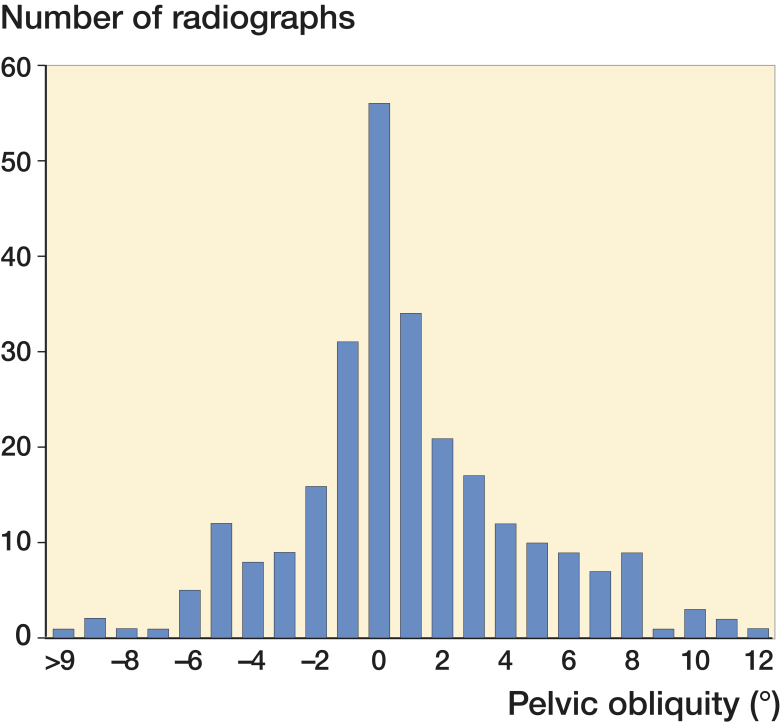
Number of pelvic radiographs related to the degree of PO. Negative value = left side of pelvis elevated, positive value = right side of pelvis elevated.

**Figure 3.**
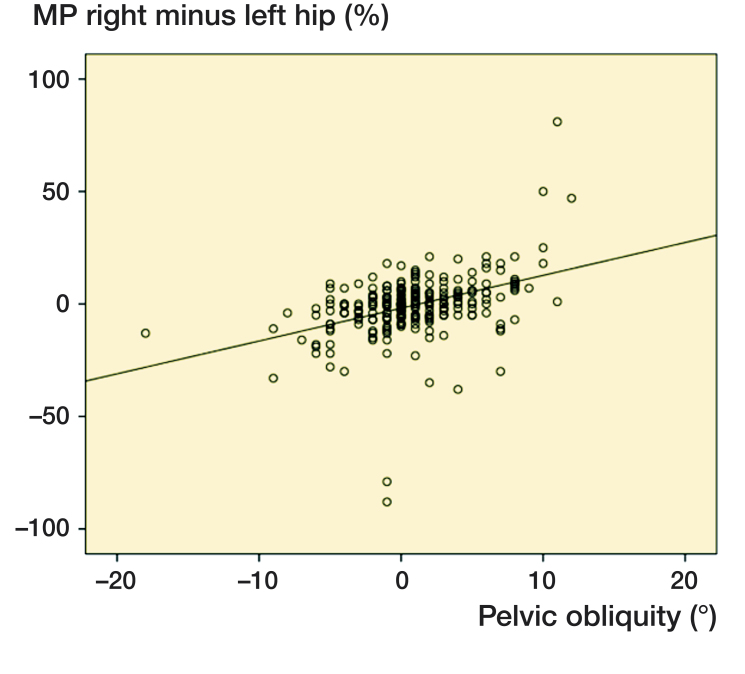
Difference between MP in the right and left hip related to PO. Negative PO value = left side of pelvis elevated, positive PO value = right side of pelvis elevated.

**Figure 4.**
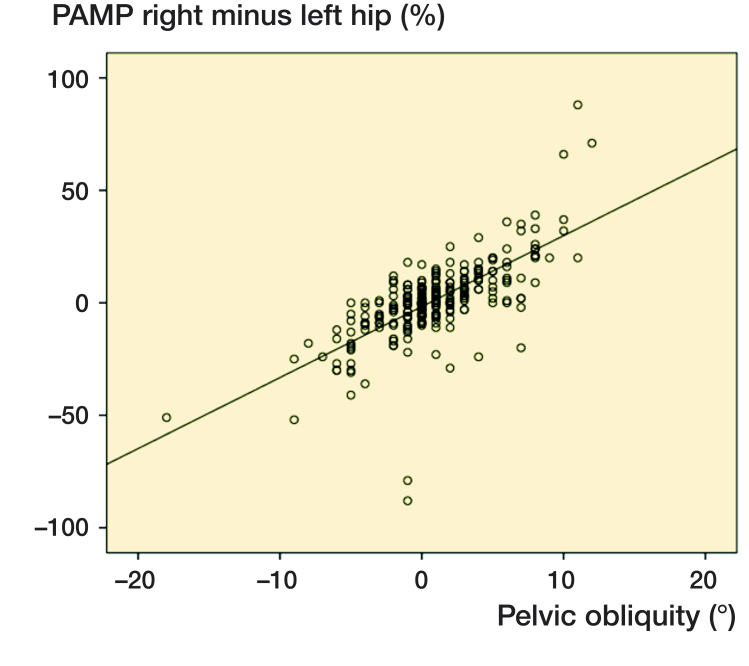
Difference between PAMP on the right and left hip related to PO. Negative PO value = left side of pelvis elevated, positive PO value = right side of pelvis elevated.

In the cohort of 268 children, the PO ranged from 0 to 18 degrees. The left side was higher in 86 cases, the right in 126, and in 56 radiographs the pelvis was neutral (Figure 2). The MP/PAMP was higher on the high side of the PO in most cases. Of the 64 radiographs with PO >5 degrees, 51 had a higher MP and 61 a higher PAMP on the high side compared with the lower side of the PO. The MP was equal on both hips in 3 cases and lower on the high side of the PO in 10 cases. The PAMP was equal on both hips in 1 case and lower in 2 cases. The difference between PAMP and MP on the high side of the PO increased with degree of obliquity (Table 3). There was a statistically significant (p < 0.001) correlation between the MP or PAMP in the right minus the left hip and the PO. Pearson’s correlation coefficient was 0.41 (CI 0.28–0.54) for the comparison with MP and 0.70 (CI 0.61–0.79) for PAMP (Figures 3 and 4).

**Table 3.** Difference between PAMP and MP on the high side of pelvic obliquity

Pelvic obliquity (degrees)	1–4	5–9	≥10
Number of radiographs	148	57	7
PAMP–MP			
mean difference (90% percentiles)	1.9 (0 to 7)	6.5 (0 to 14)	11
median difference (range)	0 (–4 to 10)	6 (–5 to 16)	9 (0 to 24)

For abbreviations, see Table 2

## Discussion

The difference between the new (PAMP) and traditional (MP) method of measuring hip displacement in CP is the use of a horizonal line instead of Hilgenreiner’s line. This study showed that PAMP had a slightly higher interrater reliability, and a substantially higher correlation with PO than the MP. When the reference points for Hilgenreiner’s line are indistinct, it is easier to draw a horizontal line.

Heidt et al. (2015) analyzed PO in 98 individuals with CP at 19 years (15–24) of age. They found a high inter- and intrarater reliability for PO measurement and a strong correlation between PO and hip morphology.

PO and PAMP depend on the position of the child on the examination table. The table is often narrow and it seems reasonable that the child is positioned in the middle line of the table. The CPUP manual states that the child should be positioned with the legs straight. The radiographic examinations in this study were performed in 11 different radiology departments. Therefore, the results are likely to represent the situation in routine care and could be generalized to hip surveillance programs in different settings.

Porter et al. (2007) found no correlation between the side of hip displacement, defined as MP >33%, and high/low side of PO in a study of 747 individuals with CP. Lonstein and Beck (1986) reported similar results with no correlation between the high side of the PO and the side of hip displacement, defined as MP >33%, and no increase in hip displacement with increasing PO among 304 individuals with CP classified as dependent sitters. In both these studies, PO was measured in the sitting position. A PO caused by asymmetric hip abduction is only present in the lying or standing position. In the sitting position, restricted abduction will rotate the pelvis backwards, or the legs will deviate into adduction, but it cannot force the pelvis into PO in the sitting position, which might explain the absence of correlation found in these studies. Letts et al. (1984) did a longitudinal follow-up study of all radiographs, both sitting and supine, from infancy to the teen ages in 22 children with hip displacement, PO, and scoliosis. All hip displacements occurred on the high side of the PO.

There are limitations to this study. This was a descriptive and psychometric evaluation of a new measurement of hip displacement based on radiographs from 1 occasion for each child. To demonstrate a stronger causal relationship between PAMP and hip dislocation than with MP and hip dislocation, a longitudinal study comparing the 2 measures as risk factors is required. However, with knowledge of the cause of hip dislocation and the force vectors of the hip joint as described by Miller et al. (1999), there is theoretical support that PAMP better reflects the risk of hip dislocation. On radiographs with no or small PO, the MP is equal to the PAMP. In higher degrees of PO, MP probably underestimates the risk of hip dislocation on the high side and overestimates the risk on the lower side. No child in this series had a PO that exceeded 18 degrees. Therefore, we cannot make conclusions on the relationship between MP and PAMP in cases with larger pelvic asymmetries.

The results of this study were based on all children with CP in the area examined in the CPUP program. However, hips operated with varus osteotomy and spinal surgery were excluded; therefore, the study was made on a selected cohort with relatively few children in GMFCS V.

In summary, measurement of hip displacement using the new PAMP showed a high interrater reliability, and a higher correlation with PO than the regular measurement of MP. The measurement of PAMP is in agreement with known consequences of forces on the hip joint and is likely to provide a more accurate reflection of the risk of hip dislocation in hips with a PO. The PAMP has the potential of being more accurate in hip surveillance. We suggest the use of PAMP instead of regular MP at least in hips with a pelvic obliquity exceeding 5 degrees.

## Study design

: GH, MG, MH, ERB. Data collection: GH. Data analysis: GH, MG, MH, ERB. Manuscript preparation: GH, MG, MH, ERB.

Acta thanks Freeman Miller and Terje Terjesen for help with peer review of this study.
